# Identification of children who may benefit from self-hypnosis at a pediatric pulmonary center

**DOI:** 10.1186/1471-2431-5-6

**Published:** 2005-04-25

**Authors:** Ran D Anbar, Susan C Geisler

**Affiliations:** 1 Department of Pediatrics, University Hospital, State University of New York Upstate Medical University, Syracuse, NY, USA

**Keywords:** anxiety, asthma, habit cough, hypnosis, vocal cord dysfunction

## Abstract

**Background:**

Emotional difficulties can trigger respiratory symptoms. Thus, children presenting with respiratory complaints may benefit from a psychological intervention. The purpose of this study was to define the proportion of patients referred to a Pediatric Pulmonary Center who may benefit from instruction in self-hypnosis, as a psychological intervention.

**Methods:**

A retrospective chart review was conducted for all newly referred patients to the SUNY Upstate Medical University Pediatric Pulmonary Center during an 18 month period beginning January 1, 2000. Patients were offered hypnosis if they presented with symptoms or signs suggestive of psychological difficulties. Hypnosis was taught in one or two 15–45 minute sessions by a pediatric pulmonologist.

**Results:**

Of 725 new referrals, 424 were 0–5 years old, 193 were 6–11 years old, and 108 were 12–18 years old. Diagnoses of anxiety, habit cough, or vocal cord dysfunction accounted for 1% of the 0–5 year olds, 20% of the 6–11 year olds, and 31% of the 12–18 year olds. Hypnotherapy was offered to 1% of 0–5 year olds, 36% of 6–11 year olds, and 55% of 12–18 year olds. Of 81 patients who received instruction in self-hypnosis for anxiety, cough, chest pain, dyspnea, or inspiratory difficulties, 75% returned for follow-up, and among the returning patients 95% reported improvement or resolution of their symptoms.

**Conclusion:**

A large number of patients referred to a Pediatric Pulmonary Center appeared to benefit from instruction in self-hypnosis, which can be taught easily as a psychological intervention.

## Background

Emotional difficulties can trigger respiratory symptoms such as dyspnea [1]. Further, psychological issues can arise as a result of patients' reactions to pulmonary disease, e.g., depression with end-stage cystic fibrosis [2]. In some patients, a vicious cycle ensues as pulmonary disease leads to psychological difficulties, which in turn trigger further symptoms that can be misinterpreted as arising from the pulmonary disease [3]. Thus, a patient with severe asthma can develop anxiety as a result of the life-threatening nature of the disease. Such stress can cause dyspnea, which might be treated inappropriately with therapy for asthma rather than anxiety [4]. In a study by Ortega et al. [5], 49% of children with asthma from cohorts in New Haven, Atlanta, NewYork, and Puerto Rico were identified through the Diagnostic Interview Schedule for Children as having an anxiety disorder. Further, a history of psychogenic stressors or psychiatric disorder often is identified in pediatric patients with functional respiratory disorders, such as sighing dyspnea, habit cough, and vocal cord dysfunction [6]. For example, in a literature review, 52% of patients with vocal cord dysfunction were diagnosed as having a conversion disorder [7]. This evidence suggests that children presenting with respiratory complaints may benefit from a psychological intervention.

Previously, we reported the outcome of hypnotherapy offered at our Pediatric Pulmonary Center [1,4,8-10]. Reported rates of improvement following hypnotherapy ranged from 86% of patients with anxiety [8], 90% of patients with habit cough [10], 91% of patients with vocal cord dysfunction [8], and 100% of patients with chronic dyspnea, who had normal lung function at rest [9].

The purpose of the current study was to define the proportion of all patients referred to our Pediatric Pulmonary Center who might receive benefit from instruction in self-hypnosis as a psychological intervention.

## Methods

A retrospective chart review was undertaken for all patients newly referred to the SUNY Upstate Medical University Pediatric Pulmonary Center during the 18 months beginning January 1, 2000. Most of these referrals were from primary care providers. Information collected included age, gender, referral diagnosis, whether and for what purpose they were offered hypnotherapy or any other psychological intervention, the results of the intervention, and diagnosis in 2003 at the time of the data collection, or at the time of discharge from the Center if this occurred before 2003. Assessment of intervention effectiveness was based on the patients' subjective reports, except in the cases of patients with habit cough, or stridor associated with vocal cord dysfunction, whose symptoms were observed to have resolved during a visit at our Center.

Patients were evaluated for their respiratory complaints by a thorough review of their history, physical examination, and laboratory investigations, including pulmonary function testing, radiological investigations, and blood studies. Patients were offered hypnosis if they presented with symptoms or signs suggestive of psychological difficulties, such as those listed in Table 1. Formal testing for psychological disorders or hypnotizability was not utilized. Those who expressed interest in hypnotherapy were instructed in self-hypnosis techniques by a pediatric pulmonologist. Hypnosis was taught in one or two 15–45 minute sessions, as described previously [8]. Patients who required psychological intervention other than hypnotherapy were to be referred to a child psychiatrist.

**Table 1:** Symptoms and signs suggestive of psychological difficulties*

Respiratory symptoms

Difficulty with inspiration
Disruptive cough
Dyspnea despite normal lung function
Hyperventilation
Inspiratory noise (e.g., stridor, gasping, rasping, or squeak)
Localization of breathing problem to the neck or upper chest
Sighing


Other symptoms

Anxious appearance
Dizziness
Feeling something is stuck in the throat
Palpitations
Paresthesias
Shakiness


Symptom characteristics

Absence during sleep or when patient is distracted
Associated with a particular location or activity
Emotional response to symptoms
Emotional trigger of symptoms
Exposure to traumatic life event
Incomplete response to medications

* Adapted from references 1, 4, 8–10.

As this study involved a retrospective chart review without identification of patients, exemption was given from review by the Institutional Review Board.

## Results

Of the 725 newly referred patients (424 were 0–5 years, 193 were 6–11 years, and 108 were 12–18 years old), 133 (18%) were offered hypnotherapy. No patients required referral to a psychiatrist. Patients offered hypnotherapy tended to be older: Hypnotherapy was offered to 1% of 0–5 year olds, 36% of 6–11 year olds, and 55% of 12–18 year old patients. Table 2 lists the main reasons for the hypnotherapy.

**Table 2:** Reasons for offering hypnotherapy

n = 133	
	Percent of patients

Respiratory symptoms	
	
Cough	19
Chest pain	8
Dyspnea	25
Inspiratory difficulties	8
	
Other reasons	
	
Altering palatability of medications	8
Anxiety	9
Headaches	6
Insomnia	4
Relaxation	11
Other	14

Anxiety, habit cough, and vocal cord dysfunction were the three diagnoses made at our Center in this study population that are recognized commonly as having major psychological components [5,6]. These diagnoses accounted for 1% of the 0–5 year olds, 20% of the 6–11 year olds, and 31% of the 12–18 year olds.

A referral diagnosis of asthma accounted for 28% of 0–5 year olds, 50% of the 6–11 year olds, and 53% of the 12–18 year olds. Among these patients, anxiety, habit cough, and vocal cord dysfunction were diagnosed in 4% of 0–5 year olds, 21% of the 6–11 year olds, and 26% of the 12–18 year old patients.

One hundred sixteen of the 133 patients (87%) offered hypnotherapy agreed to receive instruction in self-hypnosis. Eighty-one of the patients received such instruction for anxiety or the respiratory symptoms listed in Table 2. Among these 81, 75% returned for follow-up, and among the returning patients 95% reported improvement or resolution of their symptoms, as previously described in detail for many of these patients [1,8-10].

## Discussion

This study demonstrates that a large number of patients referred to a Pediatric Pulmonary Center may benefit from instruction in self-hypnosis. As reported elsewhere, many of the patients in this report, e.g., those with anxiety, habit cough or vocal cord dysfunction, failed to improve prior to introduction of hypnosis [1,8-10]. Therefore, it is likely that hypnosis was important for their recovery. It is possible that the time spent with the pulmonologist, or the reassurance received regarding the absence of physiologic disease were the critical parts of the intervention, as opposed to the hypnotherapy. Even if this were the case, the findings in this report underscore that a significant number of patients can respond to a therapeutic interaction that addresses their psychological needs.

Given our finding that a large number of referred patients may benefit from psychological intervention, we believe that health care providers should familiarize themselves with symptoms and signs that may indicate psychological difficulties (see Table 1). Further, providers should identify mechanisms by which patients' psychological issues can be addressed appropriately. Provision of instruction in self-hypnosis techniques can allow for a rapid, effective intervention that patients often accept readily [8-11]. Hypnosis should not be offered in situations where it might aggravate existing emotional problems, or the problem might be treated more effectively by another method [11]. Interested clinicians can receive training in self-hypnosis techniques through workshops sponsored by the American Society of Clinical Hypnosis, the Society for Clinical and Experimental Hypnosis, or the Society for Developmental and Behavioral Pediatrics [11].

The proportion of patients with psychological difficulties contributing to respiratory symptoms at our tertiary-care Center may be greater than that in a general pediatric practice because patients who respond well to medical therapy are less likely to be referred. Further, the proportion also may be different when compared to other Pediatric Pulmonary Centers. For example, if the socio-economic mix of referred patients is different between Centers, the proportion of patients with physiologic disease may vary between Centers as a result of different levels of environmental exposures and adherence to prescribed therapy. Also, the type of patient referred to our Center may be biased as a result of our recognized interest in the treatment of symptoms with a possible psychological basis. On the other hand, it is possible that we would have diagnosed more patients with psychological difficulties had we used formal psychological testing [5,11]. The relatively high number of patients lost to follow-up in this report is attributable to the study population that was derived from a clinical practice. Thus, controlled studies with close follow-up are needed to help better define the utility of self-hypnosis.

Psychological intervention in the comprehensive management of pediatric patients also is likely to be of benefit for a large number of patients in other pediatric practice settings including general pediatrics [11], and sub-specialty centers, including gastroenterology (e.g., for functional abdominal pain) [12], nephrology (e.g., for enuresis and dysfunctional voiding) [11], neurology (e.g., for headaches) [11], and surgery (e.g., for promotion of recovery) [11].

## Competing interests

The author(s) declare that they have no competing interests.

## Authors' contributions

RA is the pediatric pulmonologist described in this report. He conceived the study and wrote the manuscript. SG collected and analyzed the data, and revised the manuscript.

## Pre-publication history

The pre-publication history for this paper can be accessed here:


